# Cyclodextrin pendant polymer as an efficient drug carrier for scutellarin

**DOI:** 10.1080/10717544.2020.1856223

**Published:** 2020-12-13

**Authors:** Rongqiang Liao, Ying Liu, Pin Lv, Di Wu, Meiling Xu, Xiaoyuan Zheng

**Affiliations:** a Pharmacy Department, Chongqing Emergency Medical Center, Chongqing University Central Hospital, Chongqing, P.R. China; b Industrial Crop Research Institute, Yunnan Academy of Agricultural Sciences, Kunming, P.R. China; c Faculty of Life Science and Technology, Kunming University of Science and Technology, Kunming, P.R. China

**Keywords:** Cyclodextrin, poly(ε-lysine), drug carrier, scutellarin

## Abstract

A novel β-cyclodextrin pendant polymer (ε-PL-CD), composed of poly(ε-lysine) (ε-PL) main chain and glycine-β-cyclodextrin (Gly-CD) side chains, was prepared by a simple two-step procedure. The ε-PL-CD was investigated as a drug carrier of hydrophobic drug scutellarin (SCU). The characterization and complexation mode of the SCU:ε-PL-CD were researched in both solution and solid state by means of photoluminescence spectroscopy, ^1^H and 2D NMR, X-Ray powder diffraction (XRPD), thermal gravimetric analysis, Particle size and Zeta potential. The solubility test indicated that the solubilizing ability of SCU:ε-PL-CD was significantly improved compared with SCU:β-CD and free SCU. Besides, in *vitro* cell experiment, it was found that SCU:ε-PL-CD has a strong inhibitory effect on the growth and invasion of tumor cells. The present study provides useful information for ε-PL-CD as a drug carrier material.

## 1. Introduction

The design of advanced drug delivery systems with insignificant side effects toward normal tissues and high therapeutic efficacy to malignant tumors remains a major challenge in the biological materials field (van der Meel et al., 2019; Zhou et al., 2020). In the past decade, polymer drug delivery systems, which have controlled release capability, targeted delivery capability, biocompatibility, and biodegradability, have been widely researched (Jang & Sengupta, 2019). Polymers with novel architectures, such as pendant polymers, dendritic polymers, and star-shaped polymers, have attracted a great of attention, in recent years, due to their unique properties and functions, which polymers with common linear structures may not have (Sarkar & Levi-Polyachenko, 2020). Poly(ε-lysine)-MW4kD (ε-PL) is one of the most preferred starting materials for the synthesis of polymers with novel architectures. poly(ε-lysine) is a linear cationic homopolyamide of 25-35 l-lysine residues, having peptide linkage between α-carboxyl and ε-amino groups (Shih et al., 2006; Hosseinzadeh et al., 2020). Recently, poly(ε-lysine) is extensively explored in biomedical field, especially in drug and gene delivery (Tian et al., 2013; Liu et al., 2014; Lv et al., 2014; Yuan et al., 2018). Similarly, β-cyclodextrin (β-CD) has been widely used in pharmaceutical field due to their excellent performance characteristics such as low toxicity, biodegradability, stability, biocompatibility, and ease of chemical modification (Zhang et al., 2020). A variety of cyclodextrin-based biological materials such as cyclodextrin polymers, cyclodextrin polyrotaxanes, and cyclodextrin dendrimers are widely used to construct drug carriers (Alvarez-Lorenzo et al., 2017; Tian et al., 2020).

In this article, we chose β-cyclodextrin as a polysaccharide to be grafted to the poly(ε-lysine) because β-cyclodextrin is readily functionalized and because, more importantly, β-cyclodextrin can contain various poorly soluble drug molecules in its hydrophobic cavity, but the prerequisite is that the size and shape of the drug molecules have a good match with the cavity of cyclodextrin (Yang et al., 2013). The hydrophobic moiety of the drug is encapsulated in the cavity of β-cyclodextrin, which was caused by the noncovalent bond between host and guest. The hydrophobic drug molecules carrier of β-CD pendant polymer can be endocytosed by cells, which results in the internalization of the encapsulated drug. The main reason may be the cationic characteristics of the carrier.

Scutellarin (SCU, 4′,5,6-trihydroxyflavone-7-*O*-glucuronide) is widely found in Chinese herb *Erigerin breviscapus* (Vant.) Hand.-Mazz, which are naturally occurring flavones. Scutellarin has the functions of expanding blood vessels, improving microcirculation, increasing cerebral blood flow, and inhibiting platelet aggregation (Mo et al., 2018; Fu et al., 2019). In recent years, it has been discovered that scutellarin can also inhibit the main protease of SARS-CoV-2, inhibit the activity of HIV-1 reverse transcriptase and anti colon cancer pharmacological function (Zhang et al., 2005; Liu et al., 2019; Sharma et al., 2020; Yue et al., 2020). Although scutellarin is widely used, it cannot be ignored that its poor water solubility leads to low bioavailability.

**Figure 1.**
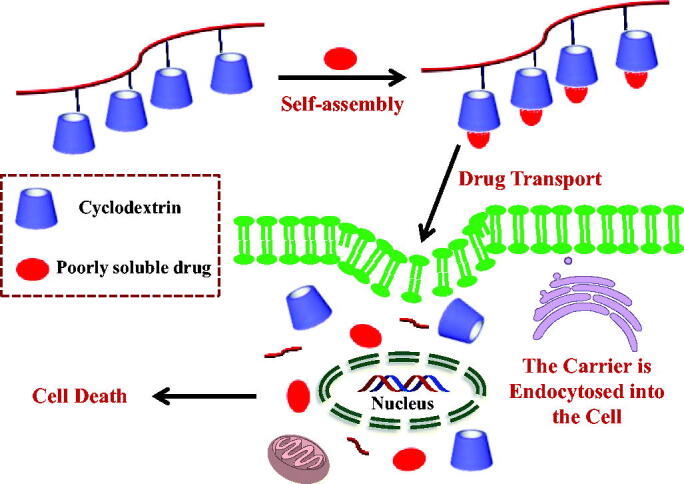
Conceptual diagram of β-Cyclodextrin pendant polymer:drug complexation.

The inclusion behavior of common cyclodextrin with scutellarin has been studied, but its solubility and bioavailability are not significantly improved, which may be due to the low drug loading. To further improve the solubility, bioavailability, and cellular uptake of scutellarin, we chose β-cyclodextrin pendant polymer as a drug carrier for research. Under the same conditions, the drug loading of newly constructed β-cyclodextrin pendant polymer is much higher than that of common cyclodextrin. The β-cyclodextrin pendant polymer can make the medicine reach the lesion at the same time (Figure 1).

## 2. Experimental

### 2.1. Materials

Scutellarin was obtained from Kunming Pharmaceutical Co., Ltd. Poly(ε-lysine) and β-cyclodextrin were commercially available. Gly was obtained from Aladdin Industrial Corporation. All solvents were of reagent grade, purchased from commercial sources, and used without further purification, which were dried with CaH_2_ under N_2_, filtered, and distilled under reduced pressure. All experiments were carried out using ultrapure water.

### 2.2. Synthesis of glycine-β-cyclodextrin

Glycine-β-cyclodextrin (Gly-CD) was synthesized according to the previous literature (Liu et al., 1999, 2003). Gly (1.0 g, 13.3 mmol) and mono-6-*O*-(*p*-tolylsulfonyl)-β-CD (6-OTs-β-CD) (6.0 g, 4.7 mmol), which was synthesized by β-cyclodextrin and *p*-toluenesulfonyl chloride in aqueous solution, were dissolved in water (48 mL) containing triethanolamine (32 mL), and the reaction mixture heated to reflux under N_2_ for 24 h. Subsequently, the solution was concentrated to about 40 mL, and then slowly poured into absolute ethanol (300 mL). A yellow precipitate formed and was collected by filtration. Finally, the precipitate was washed 3 times with absolute ethanol and dried in vacuum to obtain a yellow powder. Yield 4.4 g (62.8%). ^1^H NMR (D2O, TMS, ppm), δ: 3.14–3.28 (m, 2H, CH_2_ of Gly), 3.45–3.95(m, 42H, H2–H6 of β-CD), 4.97–4.99 (s, 7H, H1 of β-CD).

### 2.3. Synthesis of poly(ε-lysine)-glycine-β-cyclodextrin

Poly(ε-lysine)-glycine-β-cyclodextrin (ε-PL-Gly-CD) was prepared through 1-ethyl-3-(3-dimethylaminopropyl) carbodiimide hydrochloride (EDCI) and *N*-hydroxysuccinimide (NHS) assist synthesis (Yi et al., 2017). To a solution of Gly-CD (6.0 g, 5.1 mmol) in dimethylsulfoxide (25 mL) containing dimethylfomamide (5 mL) was added EDCI (1.1 g, 8.5 mmol) and NHS (0.6 g, 5.2 mmol), and the resulting solution was stirred at 0 °C in an ice bath for 2 h. In another vial, ε-PL (0.65 g, 0.16 mmol) in dimethylsulfoxide (5 mL) was stirred for 1 h, and then added to the above solution and stirring continued for 2 days. Subsequently, the reaction solution was slowly poured into acetone (300 mL). A yellow precipitate formed and was collected by filtration. The product was dialyzed (MW 8000) for 96 h to remove small molecules. The resultant solution was concentrated under reduced pressure to a few milliliters before pouring into acetone (100 mL). A fine yellow precipitate was formed and gathered by filtration. The pure ε-PL-Gly-CD was further dried in vacuum at 40 °C overnight. Yield 3.1 g (46.6%). ^1^H NMR (D_2_O, TMS, ppm), δ: 1.02–1.79 (m, 186H, CH_2_CH_2_CH_2_ of ε-PL), 4.97–4.99 (s, 68H, H1 of β-CD).

### 2.4. Degree of substitution determination

The number of grafted Gly-CDs per ε-PL was calculated by ^1^H NMR integration, and the number of grafts was defined as the degree of substitution (DS). The DS was determined by comparing the integral of the 1-H proton of β-CD and the methylene proton of ε-PL. The characteristic data of ε-PL-Gly-CD of the average DS is 10.

### 2.5. Preparation of inclusion complex

The inclusion complex of SCU:ε-PL-Gly-CD was prepared by the saturated solution method. Briefly, SCU (Mw: 462) (0.04 mM, 18.5 mg) was dispersed into 15 mL ε-PL-Gly-CD (Mw: 15730) aqueous solution (0.001 mM, 15.7 mg). After shaking for 72 h in a thermostatic water bath at 25 °C, the solution was filtered through 0.45-µm Millipore membrane, and then the filtrate was evaporated under pressure to remove the solvent and dried in vacuum to obtain the SCU:ε-PL-Gly-CD inclusion complex.

### 2.6. Preparation of physical mixture

The physical mixture of SCU and ε-PL-Gly-CD with 10:1 molar ratio was prepared by grinding in a ceramic mortar.

### 2.7. Stoichiometry

The stoichiometry for the inclusion complexation of SCU with Gly-CD of ε-PL-Gly-CD was determined by using continuous variation technique (Job’s plot) based on the differences in absorbance of SCU (Wang et al., 2014). The fluorescence spectra obtained in the buffer solution (pH 10.5, NaHCO_3_-Na_2_CO_3_) were used to determine the Job curve. The total molar concentration (i.e. [SCU] + [ε-PL-Gly-CD]/10) was kept constant 3.0 × 10^−5 ^mol/L, but the molar fraction of SCU (i.e. [SCU]/([SCU] + [ε-PL-Gly-CD]/10)) varied from 0.1 to 0.9. The ε-PL-Gly-CD containing ten CDs, so the concentration of ε-PL-Gly-CD needs to reduce 10-fold.

### 2.8. Spectral titration

The experimental procedure was carried out as follows: the buffer solution (pH 10.5, NaHCO_3_-Na_2_CO_3_) is used to prepare the ε-PL-Gly-CD solution (1.0 × 10^−4 ^mol/L). In a 10 ml volumetric flask, sequentially add 1.0 ml SCU (3.0 × 10^−4 ^mol/L) and different amounts of ε-PL-Gly-CD (0, 0.1, 0.2, 0.3, 0.4, 0.5, 0.6, 0.7, 0.8, 0.9, 1.0 mL). Then, use a buffer solution to make up the mixed solution to the mark, and ultrasonically oscillated for 20 min at room temperature. Measure the fluorescence spectrum under λex/λem = 380 nm/474 nm. All experiments were repeated three times.

### 2.9. ^1^H NMR and two-dimensional (2D) NMR

Using Bruker Avance DRX spectrometer, ^1^H NMR spectra of ε-PL-Gly-CD and SCU were obtained at 500 MHz and 298 K in D_2_O and DMSO-d_6_, respectively. The ROESY experiment was performed on the Bruker Avance DRX500 instrument. All 2D NMR experiments were performed in D2O. Before measurement, equilibrate the sample for at least 24 h.

### 2.10. XRPD

The D/Max-3B diffractometer with Cu-Kα radiation (k = 1.5460 Å, 40 kV, 100 mA) was used to obtain X-Ray powder diffraction (XRPD) patterns at a scan rate of 5°/min. Mount the powder sample on the glass sample holder and scan between 5° and 60° in steps of 2*θ* = 0.02°. Repeat the measurement for each sample.

### 2.11. Thermal gravimetric analysis (TGA)

Use Netzsch Instruments model STA449 F1 Jupiter for TGA measurement. Place the sample (5–8 mg) in Al-pan in N_2_ atmosphere, the temperature range is 30–400 °C, and the heating rate is 10 °C/min.

### 2.12. Particle size and zeta potential

The particle size and surface charge distribution of SCU:ε-PL-Gly-CD were measured at 25 °C (Zetasizer Nano ZS of Malvern company). The inclusion compound was added to 5 ml of aqueous solution, vortexed for 5 min and then allowed to stand for 30 min. Use 0.22 μm microporous membrane to remove incompatible materials. Take 1 ml of the solution in the sample cell for dynamic laser light scattering analysis to determine the particle size. The measurement wavelength is 633 nm and the angle of the scattered light is 90°. Similarly, take another solution in the standard capillary sample cell to determine the surface charge.

### 2.13. Solubilization test

The excess inclusion complex was added to 5 mL of H_2_O, and the mixture was stirred at 25 °C for 1 h in the dark. The solution was filtered through 0.45-μm pore size Millipore membrane. The concentration of the filtrate was detected by UV-Vis spectroscopy (maximum wavelength 333 nm) by a SHIMADZU UV-2550 spectrophotometer.

### 2.14. In vitro cytotoxicity studies

The cells were cultured in suspension in RPMI 1640 with 10% fetal bovine serum and seeded into the pores of 96 well culture plate at the density of 1 × 10^4^/100 μL. After pre-cultivation in 37 °C 5% carbon dioxide humidified air for 24 h, different concentrations of solutions (SCU and its inclusion complex) were added and the cultivation continued for 48 h. The cell viability was assessed by MTT assay. In short, 20 μL of MTT stock solution was added to 100 μL cell culture in a 96-well culture plate, and then incubated at 37 °C for 4 h. Subsequently, the MTT containing medium was removed by centrifugation. The precipitated formazan was dissolved in 150 μL dimethyl sulfoxide. In a 490 nm spectrometer, the results were read and the triple average was calculated.

### 2.15. Transwell assay

To evaluate the cell invasion potential, we used Transwell chambers (8 μM pore size) covered with Matrigel. In brief, HCT116 cells (4 × 10^4^ cells/well) in 100 μL of serum-free medium (containing different concentrations of SCU and its inclusion complex) were seeded in the upper chambers, and 600 μL of medium with 10% FBS was filled to the lower chambers. After 24 h of culture at 37 °C, the cells that failed to pass through the membrane were removed from the upper chambers. Besides, migrated cells were fixed with 4% paraformaldehyde and dyed with 0.1% crystal violet. Following the cells were washed with tap water and then dried before being counted employing an inverted microscope.

## 3. Results and discussion

### 3.1. Synthesis of β-CD pendant polymer

**Figure 2.**
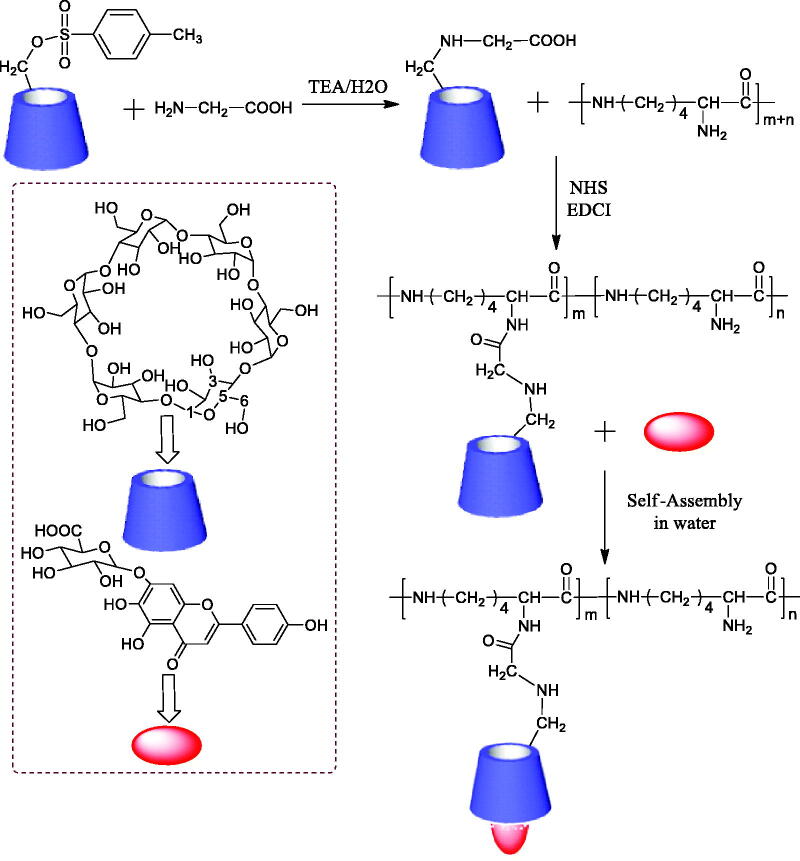
Synthetic routes for ε-PL-Gly-CD, self-assembly in water via host-guest interactions of ε-PL-Gly-CD and SUC.

**Figure 3.**
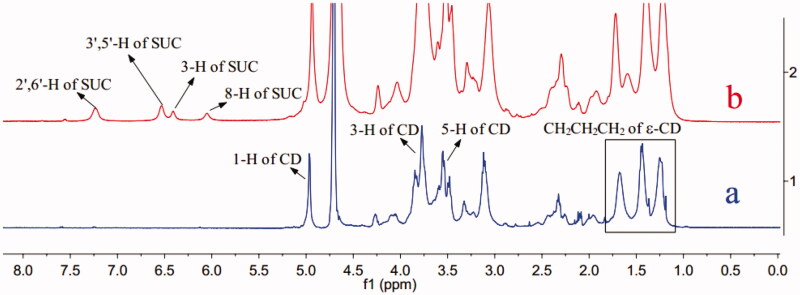
^1^H NMR spectra of ε-PL-Gly-CD (a), SCU:ε-PL-Gly-CD inclusion complex (b).

On the basis of the design shown in Figure 2, ε-PL main-chain was grafted Gly-CD through peptide linkage. The Gly-CD component was synthesized from 6-OTs-β-CD by a simple one-step procedure. The Gly-CD was further used to synthesize ε-PL-Gly-CD. The ε-PL-Gly-CD was prepared from Gly-CD and ε-PL in the presence of a catalytic amount of EDCI and NHS to give the amide-based pendant polymer. ε-PL-Gly-CD was isolated by precipitation in acetone. The number of grafted Gly-CD per ε-PL, here defined as the degree of substitution (DS), was calculated from the ^1^H NMR integration. As can be seen from Figure 3, ^1^H NMR spectra of ε-PL-Gly-CD showed the characteristic signal of 1-H protons of CD at ca. 5 ppm. The DS was determined by comparing the integrated peak area of the 1-H protons of β-CD versus that of the methylene protons of ε-PL at 0.9–1.9 ppm. The characterization data for ε-PL-Gly-CD of yield, mean DS, and mean relative molecular mass were 46.6%, 10, and 15730, respectively.

### 3.2. Stoichiometry

**Figure 4.**
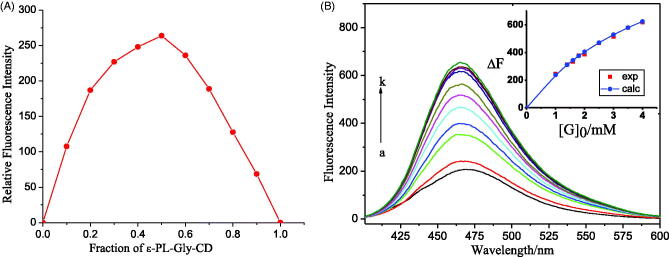
(A) Job plot for the host:guest system at λem: 474 nm ([SCU] + [ε-PL-Gly-CD]/10 = 3.0 × 10^−5^ M) in pH 10.5 buffer, (B) Fluorescence emission spectra of SCU (3.0 × 10^−5 ^mol/L) containing various concentrations of Gly-CD of ε-PL-Gly-CD (0-0.1 mM, from a to k); emission at 474 nm, and nonlinear least-squares curve-fitting analyses (insert) for the inclusion complexation.

Through the Job’s plot, the stoichiometry of the complex was calculated, starting by equimolar (3.0 × 10^−5 ^M) buffer solution of SCU and Gly-CD of ε-PL-Gly-CD, as stated in ‘Experimental’ section. Within a specific concentration range, the maximum value of the Gly-CD curve of ε-PL-Gly-CD was at a mole fraction of 0.5, which indicated that the host and guest were 1:1 bonding. (Figure 4(A)).

### 3.3. Spectral titration

The spectrophotometric titration was measured to determine the inclusion complexation behavior of Gly-CD of ε-PL-Gly-CD with SCU in arbonate buffer solution. The complex stability constant (Ks) was determined by the fluorescence intensity change caused by the addition of host Gly-CD of ε-PL-Gly-CD molecules. Since the Job’s plot showed that the stoichiometric ratio of Gly-CD of ε-PL-Gly-CD with SCU was 1:1, the inclusion complex of the host (H) and the guest (G) was represented by Equation. (1). The complex stability constant (*K_s_*) was calculated for each host–guest combination from the nonlinear squares fitting to Equation (2). Equation (2) is achieved by Equation (3).

(1) $$H+G\overset{\text{Ks}}{⇌}H\cdot G$$

(2) $$\mathrm{Ks}=\frac{\left[\mathrm{SCU}\cdot \mathrm{CD}\right]}{\left[\mathrm{SCU}][\mathrm{CD}\right]}=\frac{\Delta F/\Delta \varepsilon }{{\left([\mathrm{CD}\right]}_{0}-\Delta F/\Delta \varepsilon )({\left[\mathrm{SCU}\right]}_{0}-\Delta F/\Delta \varepsilon )}$$

(3) $$\Delta F=\frac{(\Delta \varepsilon ){\left([\mathrm{CD}\right]}_{0}+{\left[\mathrm{SCU}\right]}_{0}+1/K_{s})\pm \sqrt{{\left(\Delta \varepsilon \right)}^{2}{\left({\left[\mathrm{CD}\right]}_{0}+{\left[\mathrm{SCU}\right]}_{0}+1/K_{s}\right)}^{2}-4{\left(\Delta \varepsilon \right)}^{2}{\left[\mathrm{CD}\right]}_{0}{\left[\mathrm{SCU}\right]}_{0}}}{2}$$

Here, [SCU]_0_ and [CD]_0_ refer to the total concentration of SCU and Gly-CD of ε-PL-Gly-CD (i.e. [Gly-CD] = [ε-PL-Gly-CD]/10), respectively; Δε is the proportionality coefficient, which may be taken as a sensitivity factor for the fluorescence intensity change (Liu et al., 2002).

As illustrated in Figure 4(B), the fluorescence intensity of SCU was increased with increasing concentrations of Gly-CD of ε-PL-Gly-CD, because CD can react with solute molecules to form an inclusion complex to improve the fluorescence quantum yield. Whether the inclusion compound could be formed was mainly affected by the size, shape, and charge of the guest molecule (Liu & Chen, 2006). By using the nonlinear least square curve fitting method, the complex stability constant of the host–guest combination was obtained. Figure 4(B)(inset) shows a typical curve-fitting plot for the titration of SCU with ε-PL-Gly-CD, which shows the excellent fit between the experimental and calculated data, confirming the formation of 1:1 (i.e. SCU:Gly-CD of ε-PL-Gly-CD) inclusion complex. The results show that the *K_S_* value is 2.5 KM^−1^ at 25 °C, indicating that the host and guest are well combined.

### 3.4. Inclusion mode

By comparing the 1H NMR spectra of ε-PL-Gly-CD in the absence of SCU, the possible inclusion mode of SCU: ε-PL-Gly-CD could be speculated. As shown in Figure 3, ε-PL-Gly-CD protons displayed chemical shifts at δ1.0–5.1 ppm, which were distinct from the SCU protons (6H) (δ 6.0–8.0 ppm). After ε-PL-Gly-CD and SCU form inclusion compound, the 3-H and 5-H protons of ε-PL-Gly-CD shifted 0.04 ppm and 0.02 ppm, respectively. The 3-H protons and 5-H protons were located inside the cyclodextrin cavity at the same time, and were also close to the wide end and the narrow end of the cavity respectively. The displacement of 3-H protons was larger than that of 5-H protons, which indicated that the SCU entered the ε-PL-Gly-CD cavity from the wide end.

**Figure 5.**
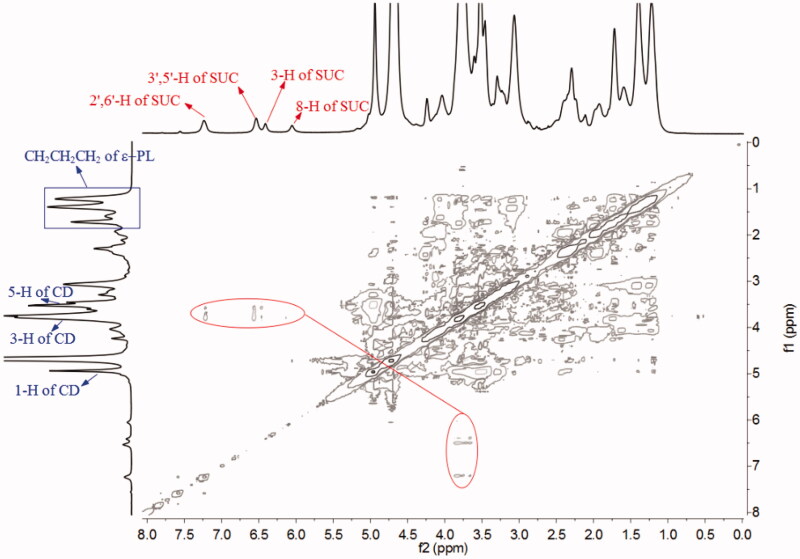
ROESY spectrum of SCU:ε-PL-Gly-CD inclusion complex in D_2_O.

**Figure 6.**
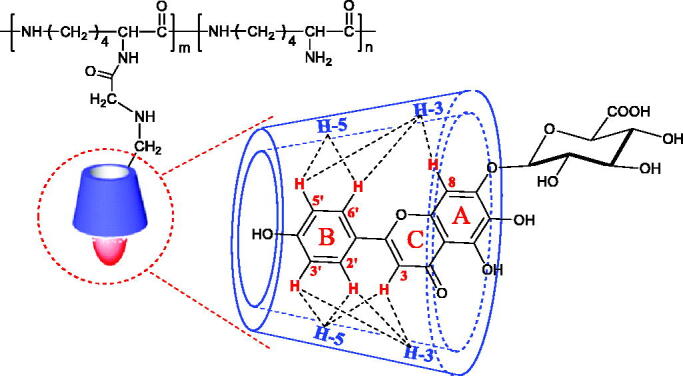
Possible inclusion mode of SCU:ε-PL-Gly-CD inclusion complex.

2D NMR spectroscopy could further infer the mode of inclusion. Protons with a distance of less than 0.4 nm in space will have cross peaks in the ROESY spectrum (Correia et al., 2002). The ROESY spectrum result of SCU:ε-PL-Gly-CD showed that the 2′,6′-H, 3′,5′-H, and 3-H protons of SCU were related to the 3-H and 5-H protons of ε-PL-Gly-CD. In addition, the 8-H proton of SCU was also related to the 3-H protons of ε-PL-Gly-CD (Figure 5). This result showed that A-ring, B-ring, and C-ring of SCU were included in the Gly-CD of ε-PL-Gly-CD cavity. According to the 1:1 inclusion stoichiometric ratio and combining these spectrum results, the possible inclusion modes of SCU: ε-PL-Gly-CD as illustrated in Figure 6 were deduced.

### 3.5. XRPD analysis

**Figure 7.**
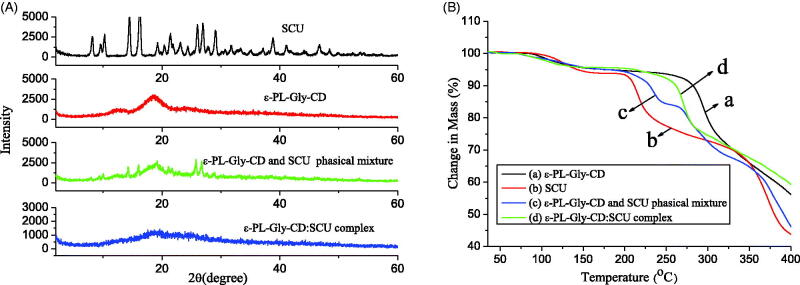
XRPD pattern (A) and TGA curve (B) of SCU, ε-PL-Gly-CD, physical mixture of SCU and ε-PL-Gly-CD, and SCU:ε-PL-Gly-CD inclusion complex.

The crystalline states of SCU, ε-PL-Gly-CD, their inclusion complex, and their physical mixture were examined by XRPD. As shown in Figure 7(A), SCU had a large number of sharp peaks, which indicated that it was crystalline. However, ε-PL-Gly-CD was relatively gentle without sharp peaks, which indicated that it was amorphous. When SCU and ε-PL-Gly-CD were physically mixed, their XRPD pattern overlapped significantly. However, an amorphous halo pattern was observed on the diffraction pattern of the SCU:ε-PL-Gly-CD inclusion compound, in which the diffraction peak of SCU disappeared completely. These results further proved that SCU was enclosed by the cavity of ε-PL-Gly-CD.

### 3.6. Thermal analysis

The thermal property of SCU, ε-PL-Gly-CD, their inclusion complex, and their physical mixture were investigated by TGA. As indicated in Figure 7(B), a systemic analysis of the TGA curves showed that ε-PL-Gly-CD decomposed at ca. 280 °C, and SCU decomposed at ca. 210 °C. In the physical mixture of SCU and ε-PL-Gly-CD, two decomposed peaks were found at ca. 220 °C and ca. 270 °C, which was the superposition of SCU and ε-PL-Gly-CD, whereas the SCU:ε-PL-Gly-CD one peak was observed at ca. 270 °C. These results proved that the SCU was encapsulated in the cavity of ε-PL-Gly-CD. Compared with the free SCU, the thermal stability of SCU after being wrapped by ε-PL-Gly-CD had been significantly improved.

### 3.7. Particle size and zeta potential

The particle size of the drug carrier is an important factor affecting drug absorption, especially for drug carriers constructed of polymer materials. The larger the particle size of the carrier, the less absorption of the drug, which in turn affects the pharmacological activity of the drug. It is found that there are three main ways for drug carriers to be absorbed: paracellular pathway (particle size <50 nm), endocytosis (particle size <500 nm) and lymphatic absorption (particle size <5000 nm) (Kulkarni & Feng, 2013). In addition, the surface charge of the carrier also affects the absorption of the drug. It is generally believed that a certain amount of positive charge on the surface of the drug carrier is easy for the carrier to be absorbed by the cell. However, the surface charge is positively related to cytotoxicity, so the positive charge on the surface of the drug carrier cannot be made too high.

**Figure 8.**
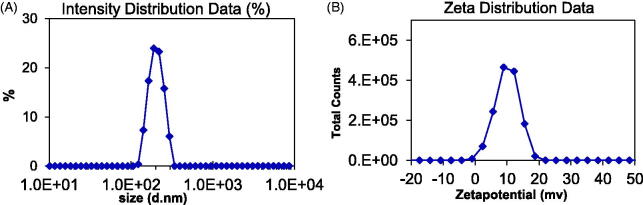
Particle size and Zeta potential of SCU: PL-Gly-CD inclusion complex.

As shown in Figure 8, the particle size of the SCU:ε-PL-Gly-CD was normally distributed, with the highest point being about 200 nm. The particle size of the assembly was just within the scale range that was easy to be endocytosed by cells. The Zeta potential of the SCU:ε-PL-Gly-CD was around 8mv, indicating that the surface of the assembly had a certain amount of positive charge. The main reason for the positive charge of the assembly was that polylysine contains a large amount of amino groups. The anionic phospholipid bilayer is a protective barrier for cells. Due to ionic interaction, carriers with weak cationic characteristics can quickly break through the phospholipid bilayer barrier and enter the cell (Bilensoy, 2010). The positive charge can further increase the cell internalization rate to the carrier. After the carrier enters the cell, the positive charge can cause the carrier to escape from the lysosome and transfer to the perinuclear area (Yue et al., 2011).

### 3.8. Solubilization

The water solubility of SCU:ε-PL-Gly-CD and SCU:β-CD was assessed by preparation of its saturated solution. An excess amount of complex was placed in 5 mL of water, and the mixture was stirred for 2 h at 25 °C. The solution was then filtered on a 0.45 µm Millipore membrane. The concentration of SCU was detected by UV-vis spectroscopy (maximum wavelength 333 nm). The result showed that the water solubility of SCU:ε-PL-Gly-CD, compared with that of free SCU (0.16 mg/mL), was remarkably increased to 52.82 mg/mL by the solubilizing effects of ε-PL-Gly-CD. However, the water solubility of SCU:β-CD was 8.74 mg/mL (Table 1). The reason for this phenomenon is probably due to the fact that the SCU:ε-PL-Gly-CD has higher equilibrium constant than SCU:β-CD inclusion complex (Yang et al., 2009). The results showed that the solubility of SCU was significantly improved after being wrapped by ε-PL-Gly-CD, which would be beneficial for the bioavailability of SCU.

**Table 1.** Concentration of SCU in different inclusion states.

	Concentration of SCU (mg/mL)
SCU	0.16
SCU:β-CD inclusion complex	8.74
SCU:ε-PL-Gly-CD inclusion complex	52.82

### 3.9. In vitro cytotoxicity studies

**Figure 9.**
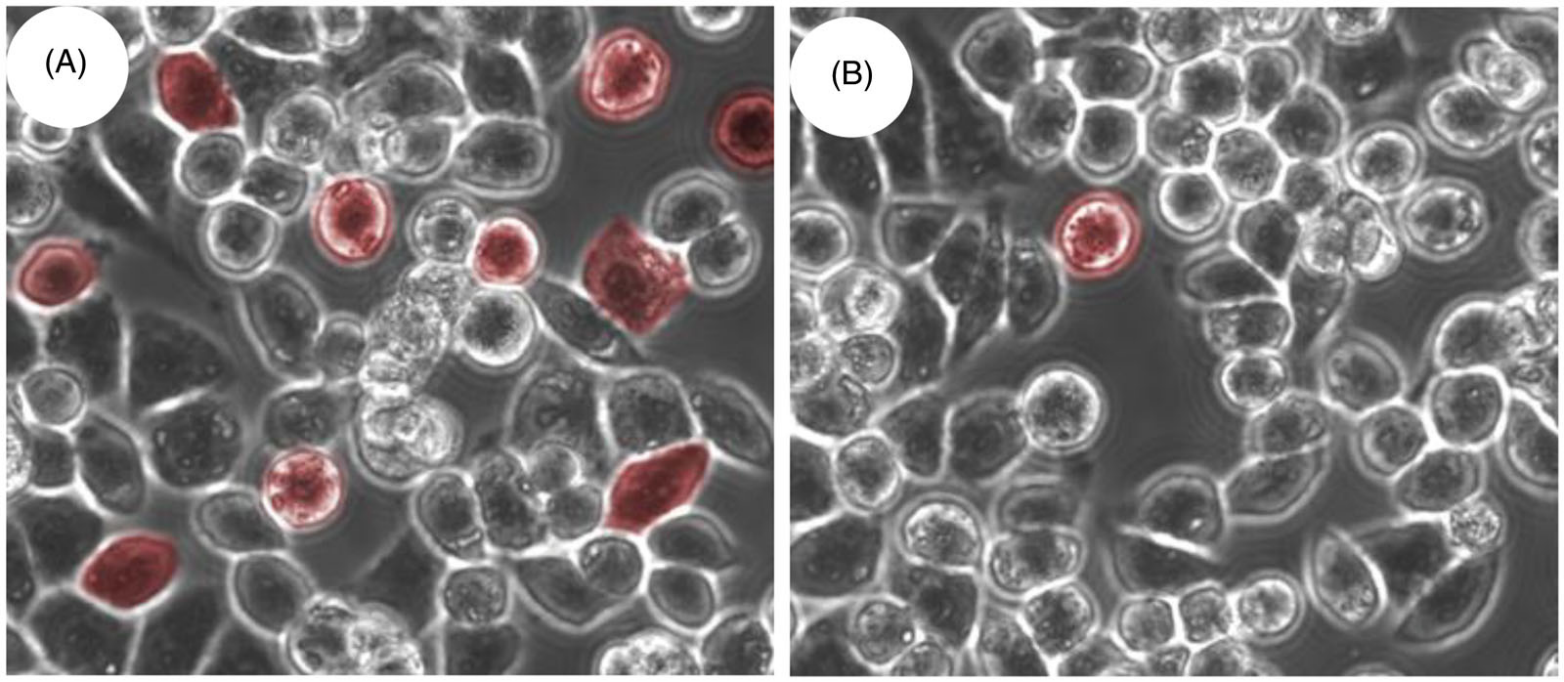
Confocal images obtained by incubation of rhodamine-B with HCT116 cells under different conditions: (A) after 4 h of incubation with rhodamine-B loaded ε-PL-Gly-CD; (B) after 4 h of incubation with rhodamine-B loaded β-CD.

Using the MTT method, the toxicity of SCU, SCU:ε-PL-Gly-CD, SCU:β-CD, and physical mixture of SCU and ε-PL-Gly-CD in HCT116 and LOVO cells were investigated separately. The IC_50_ value represented the concentration of the drug required to reduce cell growth by 50%. Table 2 showed that the IC_50_ values of the physical mixture and free SCU were no significant difference. This result indicated that ε-PL-Gly-CD had high biological safety. However, compared with free SCU and SCU:β-CD, the IC_50_ value of SCU:ε-PL-Gly-CD was obviously lower. In other words, compared with free SCU, the antiproliferative ability of SCU encapsulated in ε-PL-Gly-CD on HCT116 and LoVo cell lines was significantly improved. Figure 9 was confocal fluorescence images obtained after culturing HCT116 cells with rhodamine-B:ε-PL-Gly-CD and rhodamine-B:β-CD. The number of red cells in rhodamine-B:ε-PL-Gly-CD were significantly greater than rhodamine-B:β-CD, which showed that ε-PL-Gly-CD promoted the rhodamine-B to enter the cells. Other scholars had also reported similar observations (Salmaso et al., 2004). The outstanding results of ε-PL-Gly-CD as drug carrier are: it is grafted many CDs to which high dose of drugs can be loaded, the drug loading of ε-PL-Gly-CD is 10-fold of lone β-CD; it is easily endocytosis, which may be due to its weak cation characteristics; it has high biological safety. Furthermore, the hydrophobic drug was complexed with ε-PL-Gly-CD carrier can be released inside cells a pharmacologically active adduct that is more effective than the free drug.

**Table 2.** In vitro cytotoxic activities SCU, physical mixture of SCU and ε-PL-Gly-CD, SCU:ε-PL-Gly-CD inclusion complex, and SCU:β-CD inclusion complex.

	Anti-cancer activity IC_50_(uM)	
	HCT116	LOVO
SCU	72.3	80.6
SCU and ε-PL-Gly-CD physical mixture	78.9	77.4
SCU:ε-PL-Gly-CD inclusion complex	8.2	19.4
SCU:β-CD inclusion complex	32.8	41.3

### 3.10. Transwell assay

**Figure 10.**
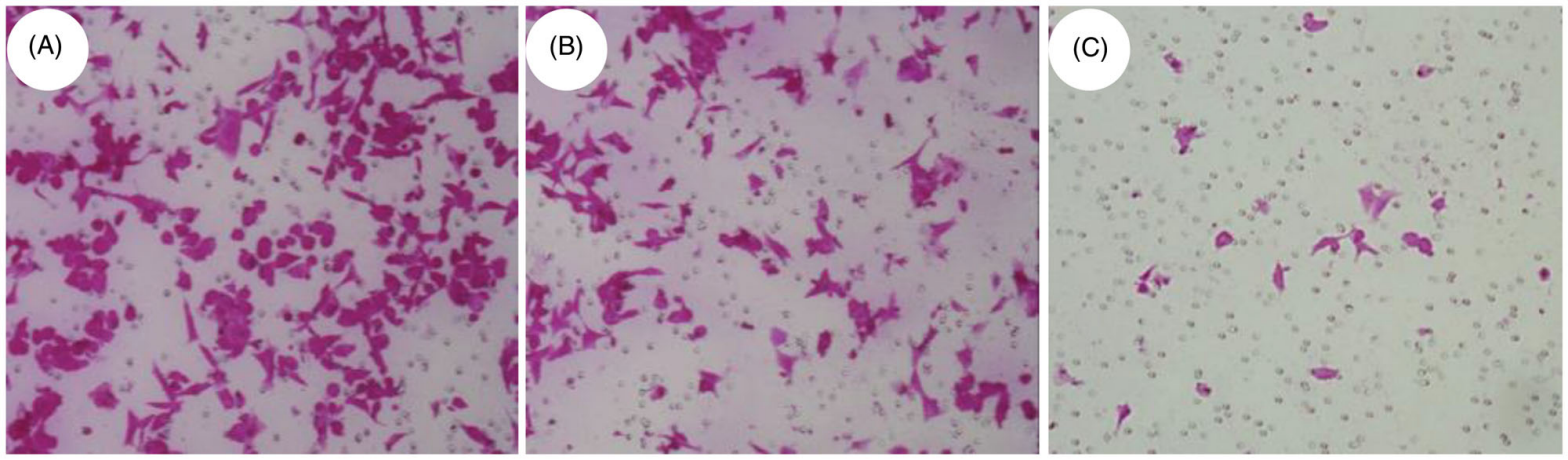
Transwell experiments were used to analyze the cell migration and invasion of HCT116 cells after the drug treatment (A: Blank, B: SCU:β-CD, C:SCU:ε-PL-Gly-CD).

The metastasis ability of a tumor is usually related to its invasion ability. The stronger the invasion ability, the stronger the metastasis ability. In the transwell assay, the greater the number of cells moving from the upper layer of the membrane to the lower layer was reported to be more invasive. Figure 10(A) shows the HCT116 cells without any treatment, a large number of cells can be found to penetrate the lower layer of the membrane. Figure 10(B) shows HCT116 cells treated with SCU:β-CD, and the number of cells penetrating the membrane was reduced compared with the blank group. Figure 10(C) shows HCT116 cells treated with SCU:ε-PL-Gly-CD, only a small number of cells passed through the membrane. The above results indicate that the invasion ability of tumor cells after SCU:ε-PL-Gly-CD treatment can be significantly inhibited.

## 4. Conclusion

In this study, we constructed a pendant polymer composed of poly(ε-lysine) main chain and cyclodextrins side chains. The inclusion complexation behavior of ε-PL-Gly-CD with poorly soluble drug SUC was investigated. These results evidence that the ε-PL-Gly-CD increased the thermal stability of SUC, and the ε-PL-Gly-CD showed superior solubilizing property for SUC in comparison to conventional β-CD. In addition, the particle size and surface charge of the inclusion compound help the drug to be endocytosed by the cell. The ε-PL-Gly-CD was tested as a delivery system for SUC in *vitro*, the SCU:ε-PL-Gly-CD can significantly inhibit tumor growth and invasion comparable to free SCU and SCU:β-CD but with no significant toxicity. In conclusion, the described ε-PL-Gly-CD provides an interesting option for drug-delivery systems.
